# Magnitude of Stunting and Associated Factors among Adolescent Students in Legehida District, Northeast Ethiopia

**DOI:** 10.1155/2021/2467883

**Published:** 2021-10-15

**Authors:** Wassachew Ashebir Kebede, Belete Yimer Ayele

**Affiliations:** ^1^Department of Public Health, College of Health Science, Debre Markos University, Debre Markos, Ethiopia; ^2^Department of Human Nutrition and Food Science, College of Health Science, Debre Markos University, Debre Markos, Ethiopia

## Abstract

**Background:**

Undernutrition including stunting particularly at an adolescent stage was not emphasized by various intervention strategies in the Ethiopian context. Assessing the magnitude and potential risk factors of undernutrition is thus helpful for policymakers to design appropriate intervention strategies. Hence, this study was aimed at assessing the magnitude of stunting and associated factors among adolescent students in Legehida district, Northeast Ethiopia.

**Methods:**

A school-based cross-sectional study was conducted among 424 adolescent students from February 15^th^ to March 15^th^, 2018. A stratified sampling followed by a simple random sampling technique was used to select the study participants. A pretested, structured, and self-administered questionnaire was used to collect the required data. Height was measured by using a portable stadiometer and the height-for-age (HFA) z-score was calculated as an indicator of stunting. SPSS version 25 and WHO AnthroPlus software were applied to analyze the data. A multivariable logistic regression analysis was performed to identify factors associated with adolescent stunting. Statistical significance was determined at a *p* value of <0.05 and association was described by using an odds ratio at a 95% confidence interval.

**Results:**

A total of 406 adolescent students (with a response rate of 95.7%) participated in the study. The magnitude of stunting among adolescent students in this study was 24.9% (95% CI: 24.6%–35.3%).

**Conclusions:**

Stunting among adolescent students was significantly associated with being male [AOR = 2.1; 95% CI: 1.73–5.90], meal frequency (<3/day) [AOR = 4.6; 95% CI: 2.61–8.24], infrequent handwashing practice [AOR = 3.6; 95% CI: 1.30–9.40], absence of latrine facility (AOR = 5.51; 95% CI: 3.03–9.9), and consumption of unsafe water [AOR = 2.8; 95% CI: 1.35–6.19]. Hence, conducting routine nutrition screenings and assessments, promotion of proper food intake, and emphasis on nutrition education and counseling are needed to be strengthened.

## 1. Background

Adolescence is a critical period of puberty characterized by substantial physical, mental, and psychosocial changes fundamentally observed which demand various nutrients. It is a time of rapid physical growth with nutritional requirements increasing significantly. In the human lifespan, it is a crucial period that provides a window of opportunity for high return on investment with nutritional interventions. Given that prevention of malnutrition in the first 1,000 days remains a priority, adolescence is another time in life that offers the last window of opportunity to break the intergenerational cycle of undernutrition [1–3]. The greater demand for energy, protein, micronutrient, and minerals because of the substantial rate of growth and development makes the period vulnerable to malnutrition. Malnutrition among adolescents includes suboptimal dietary intake of macronutrients and micronutrients as well as overweight and obesity linked to poor dietary quality [1]. Adolescents are especially vulnerable to undernutrition, in part because their rapid physical growth and development during puberty raise their nutritional needs. The absence of adequate nutrition is a risk for undermining this crucial period of growth and development. More importantly, undernutrition occurs when people do not absorb enough nutrients to cover their needs for energy, growth, and maintenance of a healthy immune system or using or excreting them more rapidly than they can be replaced [4].

It was noticed that the great majority of adolescents in the world are living in developing or emerging countries. Indeed, adolescents are increased in number than ever before and the largest increase by 2050 is expected to occur in Sub-Saharan Africa. This implies that adequate nutrition and health for this huge population group is a concern of priority. However, the health of adolescents in general and their optimal nutritional needs and services in particular remain largely neglected [5]. Besides, the current experiences and lessons learned informed that finding ways to reach the health and nutritional needs of such a large group of adolescents would remain a key challenge [6].

Undernutrition is one of the most significant universal health problems increasing the global health burden of premature mortalities and morbidities during the childhood period [7]. It is a highly prevalent health problem all over the world since up to 50% of all adolescents are stunted in some countries and numbers are significant mainly in low-income countries [8, 9]. As an important contributor to adolescent undernutrition, the habit of dietary intake by adolescents needs special emphasis. It was evidenced that the diets of adolescents in resource limited countries are generally nutritionally poor. For instance, among adolescent students, only 34% consumed fruit and 21% vegetables less than once a day [10].

The available limited evidence on adolescent undernutrition revealed that some groups of adolescents face particular challenges. In this way, the odds of undernutrition were more pronounced among adolescents of younger age and living in rural areas and male adolescents [11]. With respect to the burden of undernutrition among adolescents in different regions, there is a reported magnitude of 32–65% in Asia and 4–30% in Africa [12, 13]. With this typical vulnerability, this group is typically overlooked by, or beyond the reach of, national health, education, and development institutions. Additionally, the question of how to reach adolescent boys with nutrition interventions remains largely unanswered [14–16].

Like other low-income countries, there is no exception concerning the nutritional status of adolescents in Ethiopia. The prevalence of adolescent undernutrition in Ethiopia is very high and is increasing over time [17]. The studies conducted in Tehuledere district and Chiro Town, Ethiopia, showed that the prevalence of stunting among adolescents was 26.5% and 24.4%, respectively [18, 19]. Indeed, some other local studies have also revealed that the prevalence of stunting ranges from 12.5% to 47.4% [20]. Multiple factors influence stunting among adolescents. The sociodemographic factors are among the important determinants of stunting for adolescents [21, 22]. Additionally, reproductive health services and environmental and WASH factors were reported as contributing factors to adolescent stunting [23, 24]. According to studies done in Sub-Saharan Africa, the economy, environment, and diseases contribute to undernutrition [25].

If left unaddressed, stunting at this stage of life would have an important effect on health outcomes. Despite causing significant mortality, it results in delayed physical growth, impaired motor and cognitive development, poor concentration, decreased ability to learn and work, and lower final adult height [26]. It also leads to important consequences in adult life in terms of reproductive performance and risk of chronic diseases as malnutrition passes from generation to generation [5, 26]. In this regard, evidence-based solutions for adolescent nutritional supplementation, food system and dietary intake interventions, and integration with sexual and reproductive health strategies present crucial opportunities for improving adolescent health and well-being. Yet the scarcity of data remains a major barrier that is preventing governments from responding with effective policies, strategies, and programs. In the past decades and even today, adolescents paid little attention to nutrition-related programs mainly in developing countries including Ethiopia [2, 9].

Despite a lower mortality rate and relatively little morbidity, adolescents were typically not prioritized for targeted public health interventions. Most of the interventions conducted in Ethiopia focused on under-five children and pregnant and lactating mothers, neglecting the adolescent group in nutrition-related programs [2, 24, 26, 27]. Also, there was scanty evidence on stunting in the adolescent population mainly among student subpopulations [27, 28]. Moreover, the magnitude of stunting and factors associated with it among adolescent students were not addressed in the study area. Undernutrition including stunting particularly at an adolescent stage was not emphasized by various intervention strategies in the Ethiopian context. Assessing the magnitude and potential risk factors of undernutrition is thus helpful for policymakers to design appropriate intervention strategies. Therefore, this study was aimed at assessing the magnitude of stunting and associated factors among adolescent students in Legehida district, Northeast Ethiopia.

## 2. Materials and Methods

### 2.1. Study Setting, Design, and Period

This study was conducted in the Legehida district (administrative stage) which is located 503 km away from Addis Ababa to the north and 600 km away from Bahir Dar city in the western direction. In the district, there are two high schools with a total of 2,178 students, of which 842 are adolescent students in the age range of 10 to 19 years. According to the District Education Office 2018 report, Legehida district has a total number of 17602 students (males: 9003; females: 8599). A school-based cross-sectional study was employed among adolescent students aged between 10 and 19 years. The source population for the present study was adolescent students attending a government high school in the district, and all randomly selected adolescent students aged 10 to 19 years were the study population. Adolescent students who were severely ill and physically challenged for anthropometric measurements were excluded from the study. The data collection was conducted from February 15^th^ to March 15^th^, 2018.

### 2.2. Sample Size Determination and Sampling Procedure

A sample size of 424 adolescent students was estimated using a single population proportion formula by considering the following assumptions: a prevalence of stunting 50% (p = 0.5) (as there was no previous study on similar populations), 5% margin of error, 95% confidence level of significance (Z*α*/2 1.96), and a nonresponse rate of 10%. The two high schools, Almazbum and Shikif, were included in the study. The total samples distributed to these two schools were proportionate to their student population size. There were a total of 842 students (661 students in Shikif and 181 Almazbum high schools) whose age ranges from 10 to 19 years. Accordingly, 333 students from Shikif and 91 students from Almazbum were selected and included in the study. A stratified sampling technique was used to select the study participants, stratified based on grade level. The number of sampled students was calculated from each school and divided into grades (9^th^ and 10^th^). A sampling frame that contains the lists of high school students from grades 9 to 10 in the two schools was used based on the lists obtained from the students' record office of each school. Sample sections were selected randomly using a simple random sampling technique. Students from each section were selected again using a simple random sampling.

### 2.3. Data Collection Procedures and Instruments

Data were collected from the adolescent students using a structured and self-administered questionnaire. The questionnaire was developed based on the conceptual framework through reviewing of different literature and it covered a range of information on socioeconomic and demographic characteristics, adolescents' dietary practice, and environmental and personal hygiene of adolescents. The questionnaire was initially prepared in English and translated into the local language (Amharic) and then translated back to English to check the consistency by language experts. A total of ten data collectors with diploma holder nurses and two BSc holder supervisors participated in the data collection process. Anthropometric data were measured at the high school premise by well-trained field staff and monitored by field supervisors. Height was measured using a portable stadiometer, which consisted of an anthropometric with a simple triangular headboard to the nearest 0.1 cm based on the WHO recommendations [29]. For height measurement, two readings were recorded and the computed average was used in the analysis. WHO AnthroPlus software was applied to assess nutritional status in terms of stunting of adolescent students. Such anthropometric measurements were converted into height-for-age z-scores and compared to the new 2007 WHO reference data for 5–19 years [29, 30]. Then, the calculated height-for-age (HFA) z-score was used to classify stunting [31]. Those adolescents with height-for-age z-scores < −2SD were considered stunted. Data quality was checked during questionnaire designing, data collection, and data entry. The questionnaire was pretested among 5% of study subjects to the neighboring district (Woreilu). The data collectors and supervisors were trained at district town (Woinamba) for one day on the objectives of the study and data quality to minimize interindividual variability (measurement of precision and accuracy of each trainer were calculated and maintained during the training session).

### 2.4. Data Processing and Analysis

Before data entry and cleaning, the data were checked manually for completeness and consistency. Then, data were coded and entered into EpiData version 3.1 and exported to SPSS version 25 for analysis. Anthropometric data were entered and analyzed using AnthroPlus software. A descriptive summary (frequency with proportions, mean and standard deviations) was used to summarize the variable. Bivariable and multivariable logistic regression analyses were performed to assess the association between different independent variables and adolescent stunting. All variables with a *p* value <0.2 [31] in the bivariable analysis were entered into the multivariable logistic regression model. The odds ratio with its 95% confidence intervals was estimated to identify factors associated with stunting. A *p* value <0.05 was considered to be statistically significant.

### 2.5. Ethical Considerations

Ethical clearance of this study was approved by the Institutional Ethical Review Committee (IERC) of Health Sciences College of Debre Markos University. The official letter of cooperation was written to Legehida district health offices and a support letter from the district health office was written to high schools where the study was conducted. The nature of the study was fully explained to the study participants and parents/guardians. Well-informed verbal and written consents were obtained from the parents/guardians for adolescent students aged <18 years and assent was obtained from the participant before administering the questionnaire. Participants ≥18 years were asked to provide verbal and written consent. The collected data were kept confidential. Each participant was given a code number, and the data were stored in a secure protected place.

## 3. Results

### 3.1. Sociodemographic Characteristics of Adolescent Students

Of 424 adolescent students who participated in the study, complete data were obtained from 406 participants, making the response rate 95.8%. Of the total respondents, males accounted for 206 (50.7%). The mean age of the respondent was 16.8 ± 1.09 years. The majority of the participants, 296 (72.9%), were Muslim and all of the respondents were Amhara in ethnicity. More than half (54.2%) of the participants were living with both of their parents. The majority of the participants' families (65%) were residing in rural areas. With respect to family socioeconomic status, 32.2% and 60.3% were living in households with low and middle income, respectively. Regarding parental education, three hundred eight (75.9%) of the respondents' fathers and two hundred sixty (64%) of their mothers were literate (Table 1).

### 3.2. Water and Sanitation-Related Factors

The majority of the adolescents, 338 (83.3%), had a functional latrine at their home and 277 (68.2%) used pipe water for drinking. More than three-fourths of the adolescents, 341 (84%), had the habit of washing their hands after using the toilet, and regarding the frequency of handwashing with soap, 314 (77.3%) of adolescent students always wash their hands (Table 2).

### 3.3. Dietary and Nutritional Status of Adolescent Students

Out of the total adolescent students, 272 (67%) had a daily meal frequency of three and above. In terms of meal skipping experience, 329 (81%) of respondents were skipping their meal, and snack was the major type of meal skipped, 214 (52.7%). Based on the findings of the study, the magnitude of stunting among adolescent students in this study was 24.9% (95% CI: 24.6%–35.3%). More boys than girls were stunted (33% vs. 16.5%) in this study.

### 3.4. Factors Associated with Stunting of Adolescent Students

After controlling for the effects of potentially confounding variables using multivariable logistic regression, male sex, frequency of food intake per day, availability of latrine at home, frequency of washing hands with soap after toilet, and source of drinking water significantly predicted stunting among adolescent students (*p* < 0 : 05).

Accordingly, male adolescent students were more than 2 times more likely to be stunted than their female counterparts [AOR = 2.1; 95% CI: 1.73–5.90]. Also, adolescents who consumed food two or fewer times per day were 4.6 times more likely to be stunted than those who consumed food more than two times per day [AOR = 4.6, 95% CI: 2.61–8.24]. A significant association was also observed between stunting and availability of latrine, in which adolescents from families who did not have latrine were more than 5 times more likely to be stunted than those who had latrine at home [AOR = 5.51, 95% CI: (3.03–9.9). The frequency of washing hands with soap after the toilet was another significant factor for stunting, in which adolescents who sometimes wash their hands with soap after toilet were 3.6 times more likely to be stunted compared to those who always wash their hands [AOR = 3.6, 95% CI: (1.30–9.40)]. The odds of having stunting were almost 3 times higher among adolescents who get their water from river as compared with those who get it from pipe water source water [AOR = 2.8; 95% CI: 1.35–6.19] (Table 3).

## 4. Discussion

This study tried to determine the magnitude of stunting and associated factors among adolescent students. Accordingly, the magnitude of stunting was 24.9% (95%CI: 24.6–35.3). Male sex, daily meal frequency of less than three, infrequent handwashing practice with soap after toilet, absence of latrine facility at home, and consumption of unsafe water were significantly associated with stunting among adolescent students.

The magnitude of stunting in this study was almost comparable to a study conducted in Tigray (26.5%) [32] and Gondar town (27.5%) [33]. The possible explanation for such comparable findings could be shared social and cultural contexts, feeding experiences, economic opportunities, and degree of understanding about the advantage of optimal nutrition during adolescence. Nevertheless, it is much greater than the study conducted in Addis Ababa (7.2%) [34] and South-Western Nigeria (15.7%) [35]. This variation might be related to differences in the extent of awareness among Addis Ababa and Legehida district mothers because the relevance of optimal feeding practice and attention given to adolescents' nutrition by their families is likely to be different and better in Addis Ababa than Legehida district. Moreover, access to health care which is greatly influenced by income status and availability of high-quality foods generally can explain the existing difference in magnitude of stunting. The discrepancy with the Nigerian study is probably due to the marked variation in the habit of food intake, socioeconomic status, and cultural variation between research respondents. In Nigeria's study, most of the study participants were from urban residents that would impose less risk to develop stunting associated with better dietary practice, minimal workload, and awareness about feeding.

In this study, adolescent boys had 2.1 times higher odds of stunting compared to girls. This result was in line with the study findings in different parts of Ethiopia and Nigeria [19, 25, 35]. The reason for the high prevalence of stunting among males than females might be related to biological, behavioral, and sociocultural mechanisms. In Ethiopia, national nutritional programs and interventions had a special interest and focus on adolescent girls [36]. However, the current study shows that, compared to girls, boy adolescents are significantly being affected by stunting. Hence, nutritional programs and interventions should also give at least equal attention to boys.

Several studies around the world indicated the association between the frequency of dietary intake and the nutritional status of an individual [19, 25, 35]. In the same way, adolescents who had a meal frequency of two or fewer per day had increased odds of stunting than those reporting a higher frequency per day. The likely explanation for this association is that infrequent intake of food is not sufficient enough to meet the nutritional requirement. This might also be because skipping meals leads to inadequate dietary intake. The adolescence period has the fastest growth and the nutritional requirements are increased to promote this growth spurt. Therefore, in addition to the increased nutritional demand during the adolescence period, skipping meals leads to being stunted. Adequate meal frequency indeed accelerates a linear growth of adolescents by sufficiently supplying essential nutrients for their body size.

This study found that the absence of a latrine facility at home was significantly associated with stunting among adolescent students or they were more likely prone to undernutrition. This finding is consistent with the study performed in the East Wollega Zone [37]. This might be due to the absence of a latrine facility which leads to open defecation, increases diseases transmission, and affects nutritional status directly or indirectly. It is also a fact that proper sanitation can reduce stunting by preventing diarrheal and parasitic diseases. However, an opposite finding has been reported in Adwa [38]. The findings of this study indicated that the frequency of handwashing with soap after the toilet was significantly associated with stunting. This finding is in line with the findings from Dangla [39], East Wollega Zone [37], and Tehuledere district [18]. This might be due to irregular handwashing practice after the toilet which can cause exposure to disease occurrence and repeatedly being affected by different infections which alter the health of the adolescents.

This study found that adolescents who were using unprotected water sources (from the river) were more likely to be stunted than their counterparties. This finding is consistent with studies done in the Somali region [40], Tehuledere district [18], and Adwa [38]. This might be due to the consumption of impure water which is a vehicle for intestinal parasites (water-borne diseases) and causes loss of appetite and hence poor nutritional status directly or indirectly. Therefore, diarrhea and water-borne diseases caused by unsafe drinking water at the households' level might increase the prevalence of malnutrition directly or indirectly.

## 5. Conclusion

The finding of this study revealed that stunting among adolescent students is relatively high. Male sex, daily meal frequency of less than three, absence of latrine facility, infrequent handwashing practice, and unsafe source of drinking water were the factors independently associated with stunting among adolescent students. It is essential to increase the nutritional knowledge of adolescent students and their families to improve their nutritional intake and tackle the intergenerational effect of adolescent malnutrition. Hence, conducting routine nutrition screenings and assessment, promotion of proper food intake, and emphasis towards nutrition education and counseling are needed to be strengthened. The health extension workers should be aware of and implement sanitation and hygienic practices at the household levels. It is also crucial to avail latrine at the household level and improve the practice of handwashing before and after doing different activities. A school-based nutritional program might be helpful to reduce stunting in this group of adolescent students.

## Figures and Tables

**Table 1:** Sociodemographic characteristics of adolescent students in Legehida district, Northeast Ethiopia, 2018.

Variable	Category	Number	Percentage
Sex	Female	200	49.3
	Male	206	50.7
Age	14–16	368	90.6
	17–19	38	9.4
Religion	Orthodox	110	27.1
	Muslim	296	72.9
Living condition	With father and mother	220	54.2
	Father only	32	7.9
	Mother only	38	9.4
	With others	116	28.6
Family residence	Rural	324	79.8
	Urban	82	20.2
Family socioeconomic status	Low	131	32.2
	Middle	245	60.3
Father education	Illiterate	98	24.1
	Literate	308	75.9
Mother education	Illiterate	146	36.0
	Literate	260	64.0
Grade level	9^th^	262	64.5
	10^th^	144	35.5

**Table 2:** Water and sanitation-related characteristics of adolescent students in Legehida district, Northeast Ethiopia, 2018.

Variables	Category	Frequency	Percentage
Availability of functional latrine at home	No	68	16.7
	Yes	338	83.3
Source of drinking water	Pipe and protected spring	277	68.2
	River	129	31.8
Frequency of handwashing with soap after toilet	Sometimes	92	22.7
	Always	314	77.3

**Table 3:** Bivariable and multivariable logistic regression analysis on factors associated with stunting among adolescent students in Legehida district, Northeast Ethiopia, 2018.

Variables	Categories	Stunting		COR 95%CI	AOR 95%CI
		Yes	No		
Sex	Male	68 (33%)	138 (67%)	2.5 (1.65–4.14)	**2.1 (1.73–5.90)** ^*∗*^
	Female	33 (16.5%)	167 (83.5%)	1	1
Frequency of food intake per day	≤2 times	64 (48%)	70 (52%)	5.8 (3.63–8.99)	**4.6 (2.61–8.24)** ^*∗*^
	>2 times	37 (13.6%)	235 (86.4%)	1	1
Frequency of washing hands with soap after toilet	Sometimes	51 (55.4%)	41 (44.6%)	6.57(1.7–11.00)	**3.6 (1.30–9.40)** ^*∗*^
	Always	50 (15.9%)	264 (84.1%)	1	1
Availability of functional latrine	No	42 (61.7%)	26 (38.3%)	7.63(4.59–11.4)	**5.51(3.03–9.9)** ^*∗*^
	Yes	59 (17.4%)	279 (82.6%)	1	1
Source of drinking water	River	64 (49.6%)	65 (50.4%)	6.38(3.4–8.9)	**2.8(1.35–6.19)** ^*∗*^
	Pipe and protected	37 (13.4%)	240 (86.6%)	1	1

^*∗*^Statistical significant (*p* value <0.05).

## Data Availability

The data will be available upon request from the corresponding authors.

## Abbreviations

AOR: Adjusted odds ratio
CI: Confidence interval
COR: Crud odds ratio
HAZ: Height-for-age z-scores
OR: Odds ratio
SPSS: Statistical Packages for Social Sciences
WHO: World Health Organization.
